# Dual Arms of Adaptive Immunity: Division of Labor and Collaboration between B and T Cells

**DOI:** 10.1016/j.cell.2019.08.022

**Published:** 2019-09-10

**Authors:** K. Christopher Garcia

**Affiliations:** 1 Howard Hughes Medical Institute, Departments of Molecular and Cellular Physiology and Structural Biology, Stanford University School of Medicine, Beckman B171B, 279 Campus Drive, Stanford, CA 94305, USA

## Abstract

This year’s Lasker Basic Medical Research Award honors Max Cooper and Jacques Miller for discoveries that revealed the organizing principles of adaptive immunity. Their collective contributions have had broad clinical impact in the treatment of immune disease.

That the adaptive immune system is composed of two principal arms that defend the body from infections and cancer has been such dogma in the field of immunology for almost 50 years that it’s easy to take for granted how we came to know this as fact. Indeed, this conceptual understanding is so engrained that it transcends the specialized field of immunology to a broader societal awareness. B and T cells have become part of our popular cultural lexicon about human physiology and are often discussed in mass media in relation to public health problems such as cancer, infectious disease, autoimmunity, vaccines, and cancer therapy. It is often taught at the level of high school biology classes that there is a division of labor between B lymphocytes that mediate humoral immunity against circulating pathogens such as viruses and toxins and T lymphocytes that mediate cellular immunity against intracellular pathogens such as bacteria and fungi, virus-infected cells, and cancers and also in the rejection of transplanted tissue. The diversity inherent in each class of immune cell, now known to be due to somatically recombined antigen receptors, and antibodies, expressed by T and B cells, respectively, enables both branches to “adapt” to challenge by virtually any antigenic determinant. This is in contrast to our “innate” immune system which uses hard-wired pattern-recognition receptors that do not genetically adapt to the insult to engage infections in a first line of defense.

Max Cooper and Jacques Miller defined our contemporary understanding of the duality of adaptive immunity (Figure 1): its anatomical origins, how two separate lineages develop, how T and B cells collaborate for effective immune responses, how they contribute to host defense, how they communicate in initiating and regulating immune responses, and how defects in their development give rise to specific forms of immunodeficiency and cancers. The implications for global human health, and society as a whole, have been profound and far reaching. However, as with nearly all foundational discoveries, the path to this understanding was illuminated by clues laid by their scientific predecessors.

One of the earliest hints about the bipartite nature of adaptive immunity was made by Merrill Chase, who was working with Karl Landsteiner (who was awarded the Nobel Prize in 1930 for the discovery of human blood groups) at the Rockefeller Institute in the early 1940s. Chase observed that different kinds of immune reactivity could be transferred from one animal to another by either serum alone, containing antibodies, but no cells, or the small lymphocyte-containing “sticky exudate” fraction of the blood. At the time, it was thought that antibodies, or other chemical substances in the bloodstream, were the principal mediators of immunity. However, Chase showed that passive transfer of white blood cells could sensitize an animal to the original sensitizing chemical even in the absence of detectable antibody production in a process called contact allergy or delayed-type hypersensitivity. This was a landmark discovery of a new class of immune response termed “cell-mediated immunity”; however, it remained controversial whether or not this was truly an antibody-independent process (Chase, 1985). Chase’s observations did not postulate a division of labor between cellular and humoral immunity and left unresolved the question of where antibodies came from, the relation between antibodies and white blood cells, and how animals specifically developed and adapted their immune responses to different kinds of pathogens.

Another important foundation for Cooper and Miller’s work was clonal selection theory in the mid-1950s, originally advanced by Frank Burnet at the Walter and Eliza Hall Institute (WEHI) of Medical Research in Melbourne and David Talmage at the University of Chicago and later experimentally proven by Gus Nossal and Josh Lederberg, also at the WEHI (Nossal and Lederberg, 1958). In this model, it was hypothesized that each type of lymphocyte was clonally unique yet existed within a highly diverse pool of lymphocytes with enough reactivity bandwidth to bind to most foreign antigens, and engagement of an antigen triggers proliferation of that cell to manifest as an immune response and an immunological memory. Burnet then went on to work with Peter Medawar to refine this idea and develop concepts about how our immune system avoids reacting with the host through an early selection process called self-tolerance, for which Burnet and Medawar were awarded the Nobel Prize in 1960 (Science, 1960). Yet while these discoveries established some basic conceptual frameworks for immunology, they fell short of a clear understanding of the anatomical origins of the cells and the cellular mechanisms that orchestrate the immune response.

As a pediatrician, Max Cooper hypothesized that differences in the clinical manifestations among patients suffering from primary immunodeficiencies were due to functionally different cell lineages in the adaptive immune system, so he set out to explore this question in the laboratory. For example, patients with X-linked agammaglobulinemia controlled viral infections, so their cell-mediated immunity was intact, but they could not make antibodies, whereas patients with the X-linked disorder Wiskott-Aldrich syndrome (WAS) developed herpes infections but still could make antibodies. These paradoxical findings were difficult to rationalize in the context of a single-immune-cell lineage model. Cooper worked with Robert A. Good at Minnesota, building on earlier findings from Bruce Glick that removal of the hindgut lymphoid organ called bursa of Fabricius from chickens impaired antibody production in chickens (Glick et al., 1956). However, the problem with these experiments was that some residual cells had exited the bursa prior to removal, making these experiments, as well as contemporaneous experiments by Jacques Miller on thymus removal, difficult to reproduce and interpret. A technical improvement was needed to completely eliminate lymphocytes produced during embryonic life before bursal or thymic removal. Cooper made a key technical insight that was game changing: he irradiated the chicks soon after removing the organs in order to completely eliminate any immune cells that the chick’s thymus or bursa made before hatching. The results were then extremely clear and definitive (Cooper et al., 1965, 1966): the irradiated chickens with no bursa did not have plasma cells or produce antibodies when injected with foreign proteins, yet they had many lymphocytes. Conversely, the thymectomized and irradiated chickens were deficient in “small” lymphocytes yet retained the ability to produce antibodies. Irradiated animals that were both thymectomized and bursectomized had combined cellular and humoral immune deficits. Follow-up studies indicated that the thymus was essential for development of the small lymphocytes, later called T cells, that mediate allograft rejection, delayed-type hypersensitivity, and graft-versus-host reactions, all of which are mediated by cellular immunity, while the bursa-derived large cells, later called B cells, were required for antibody responses. The discovery of the separate thymic and bursal systems was more or less concurrent with Jacques Miller (Miller, 1961a) for the thymus in mice (with contributions from others). This two-lymphocyte model helped to explain Cooper’s original clinical observations that either cellular or humoral immunity can be defective while leaving the other system functional.

Cooper made another major finding in discovering antibody “class switching” with University of Alabama at Birmingham (UAB) graduate student Paul Kincade (Kincade et al., 1970). During an antibody response, the constant region of the immunoglobulin heavy chain changes, but the variable regions, and therefore antigenic specificity, stay the same. Cooper found in chicken B cells that IgM was expressed first, followed by switch to IgG. This seminal discovery was followed by a series of classic papers on other isotypes, including IgA, which was similarly shown to be dependent on IgM precursor cells, but not on IgG-producing cells. This finding is another pillar of adaptive immunity and highlights the essential plasticity of the immune response.

Cooper also made seminal contributions to understanding the ontogeny and stages of B cell development by identifying the equivalent organ to the bursa in mammals. He worked with Martin Raff and John Owen at University College London to identify the bone marrow and fetal liver as the origins of B cells and the precursors of B cells (Owen et al., 1974); these findings were contemporaneous with those of Gustav Nossal at the WEHI and Pierre Vassili at the University of Geneva, Switzerland, who were able to generate B cells from mouse bone marrow. After refining models of normal B cell development in humans, Cooper used this information to describe the different stages at which B cell differentiation was aborted in patients with antibody deficiencies and lymphoid malignancies. For example, that X-linked agammaglobulinemia and related B cell disorders are manifestations of arrests at distinct stages of B cell ontogeny. This understanding of the divergent lymphocyte lineages started to change the treatment of leukemias and lymphomas, as the cells of origin of these tumors can now be used to classify the cancers and to tailor therapies. This work has also established principles for grafting hematopoietic tissues that helped set the stage for the bone-marrow and stem-cell transplants that are now in routine clinical use.

In an amazing capstone to his career, Cooper became interested in the evolution of the adaptive immune system (which is characteristic of all vertebrates) and began to look in jawless vertebrates (agnathans) like the sea lamprey and hagfish, that are able to resist infection yet show no evidence of antibody production or MHC recognition. In a spectacular series of papers, Cooper found that jawless vertebrates use variable lymphocyte receptors (VLR) comprised of leucine-rich-repeat (LRR) segments as structurally distinct counterparts to the immunoglobulin-based receptors that jawed vertebrates use for antigen recognition (Pancer et al., 2004; Guo et al., 2009). Highly diverse VLR genes are somatically assembled by the insertion of variable LRR sequences into incomplete germline VLRA and VLRB genes with further diversification involving cytosine deaminases. In sea lampreys, VLRA and VLRB receptors are expressed by separate lymphocyte populations that are the functionally equivalents of T-like and B-like cells. The broad impact of these studies is the demonstration that the division of function and organ of origin of the adaptive immune system pre-date the choice of molecules that can serve as antigen receptors.

While Cooper’s work has been more B cell focused, Jacques Miller has had an equally profound impact on the field of immunology and essentially founded the field of T cell biology. Miller is among a long list of great Australian immunologists who made seminal discoveries at the WEHI (and other Australian institutions), establishing a tradition of excellence in Australian immunology that persists to this day and that has been acknowledged by many previous prizes and awards. Miller has the distinction of being the only living scientist to have discovered the function of a human organ, the thymus, as the origin of T cells. The thymus atrophies later in life and was thought to be a non-essential vestigial organ; in fact, it was routinely discarded during some forms of cardiothoracic surgery. As a graduate student, Miller became interested in immunology based on a curiosity about the origins of virus-induced leukemia in mice. He noticed that leukemias in mice started in the thymus and then spread to peripheral organs, so he wanted to see if removal of the thymus prevented leukemia. Miller developed the surgical skill to carry out a neonatal thymectomy, which no one else could do, and discovered that animals subjected to thymic ablation at birth did not develop a virus-induced leukemia and that transplanting the virus-infected thymus back into the mouse initiated the leukemia.

Importantly, the mice without a thymus were also deficient in the small lymphocytes (T cells), were unable to make antibodies (Miller, 1961b), were unable to reject transplanted foreign tissues (i.e., thymus derived cells were required graft rejection), and after several months, succumbed to many types of infections through a wasting syndrome (Miller, 1961a). Remarkably, these were single-author papers where Miller did the experiments himself, without the use of modern technologies such as flow cytometry (FACS) or knowledge of surface antigens to track cells in the blood. Miller hypothesized that the thymus was the source of cells, not hormones (as many thought), mediating cellular immunity, which was controversial, because many thought that the spleen and lymph nodes were the source of such cells. Transplanting a thymus back into these mice restored immunity by export of thymus-derived cells that seeded peripheral lymphoid tissues, which he found using chromosomal markers in the transplanted cells. Broad acceptance of Miller’s findings was hampered at the time, even though Miller was ultimately correct, because very few other labs could successfully execute the surgical technique and therefore had trouble to reproducing his findings, and also, the thymus was already producing immune cells before he removed it. Miller later began to use irradiation, as well as complete thymic ablation, which allowed other labs to reproduce his experiments. Later, in the 1970s, the advent of the “nude” mouse by other groups, in which a genetic mutation results in essentially athymic mice, further confirmed Miller’s conclusions by independent means. Nevertheless, the techniques that Miller developed to carry out his experiments, including thymectomy, lymphocyte reconstitution, and bone-marrow chimeras, are standard tools of immunology to this day and are essentially the concepts behind bone-marrow transplants carried out in the clinic.

From the original thymic ablation studies, Miller made another profound observation. He found that even MHC-mismatched thymic grafts restored immunity but were also tolerant of skin from mismatched donors, which was the first demonstration that the thymus carried out a selection step to ensure self-tolerance (Miller, 1962). Miller suggested, correctly, that lymphocytes generated in the thymus were specially selected to be tolerant to self-antigens, and that self-reactive lymphocytes were eliminated in the thymus. This was the origin of our now fundamental concepts about how immune tolerance is established.

Following up on experiments previously performed in birds by Cooper and colleagues, Miller and his colleagues demonstrated that mouse lymphocytes can be separated into two developmentally and functionally distinct lineages that were later called T and B lymphocytes, and that T lymphocytes are produced by the thymus and B lymphocytes in the bone marrow (Miller and Mitchell, 1968). Miller also observed that complete immune reconstitution, including antibody production, required a thymus (which was important to the subsequent successful medical development of allogeneic bone transplantation). With his student Graham Mitchell at the WEHI, Miller showed that thymus-derived lymphocytes were not actually making Abs yet were necessary for it (Mitchell and Miller, 1968). Miller conclusively showed that bone marrow-derived cells were responsible for Ab production, but the thymus-derived cells were absolutely essential in order to allow the bone marrow-derived cells to make antibodies, so there must be an interaction between thymus derived cells and bone marrow-derived cells (Miller and Mitchell, 1967). These results really began to shape our understanding of the interactions of immune cells and the concepts of immune regulation to “help” or suppress immune response in an antigen-specific manner.

Collectively, Miller established the main tenets of T cell biology: that the thymus not only is a source of T cells, but also is the place where undifferentiated stem cells are “educated” for self-tolerance and acquire immunocompetence through interaction with thymic epithelial cells; that T cells mediate allograft rejection, and inhibiting T cells is therapeutically useful in transplantation; that T and B lymphocytes are different lineages of cells that achieve their fate by developing in different organs; and, finally, that effective immunity is derived from both a division of labor and collaboration between T and B cells. Jacques Miller essentially founded the field of T cell biology; there are now at least six or seven different known T cell subsets, such as regulatory T cells (Tregs), Th1/Th2, Th17, and others. Clinically, Miller’s work set the stage for our understanding of autoimmunity and tolerance, as well as recent development of T cell-based immunotherapies–particularly adoptive cell therapy using chimeric antigen receptors (CAR-T) and tumor infiltrating lymphocytes (TILs).

The legacy of Cooper and Miller’s discoveries have reverberated through both basic and translational immunology for over 50 years. Cooper and colleagues’ discoveries about B cell biology led to the invention of hybridomas (Kohler and Milstein, Nobel Prize 1984), as well as understanding how antibody clonal diversity is generated (Tonegawa, Nobel Prize 1976). Miller’s work set the stage for subsequent discoveries on MHC restriction (Baruj Benaceraff, Nobel Prize 1980; Rolf Zinkernagel and Peter Doherty, Nobel Prize 1996), the isolation and cloning of T cell receptors (Mark Davis, Tak Mak, John Kappler, Pippa Marrack, and others), antigen presentation (Emil Unanue, Jack Strominger, and Don Wiley, Lasker Basic Medical Research Award 1995), and immune regulation (Ralph Steinman, Lasker Basic Medical Research Award 1995, Nobel Prize 2011).

Cooper and Miller’s discoveries fueled rapid advancements in both basic and applied sciences, creating ripple effects socially and economically. These recent accomplishments would not have been possible without the ceaseless efforts in basic immunology and translational research that catalyzed by the pioneering discoveries made by Cooper and Miller, for which they richly deserve the 2019 Lasker Award for Basic Biomedical Research.

## Figures and Tables

**Figure 1.**
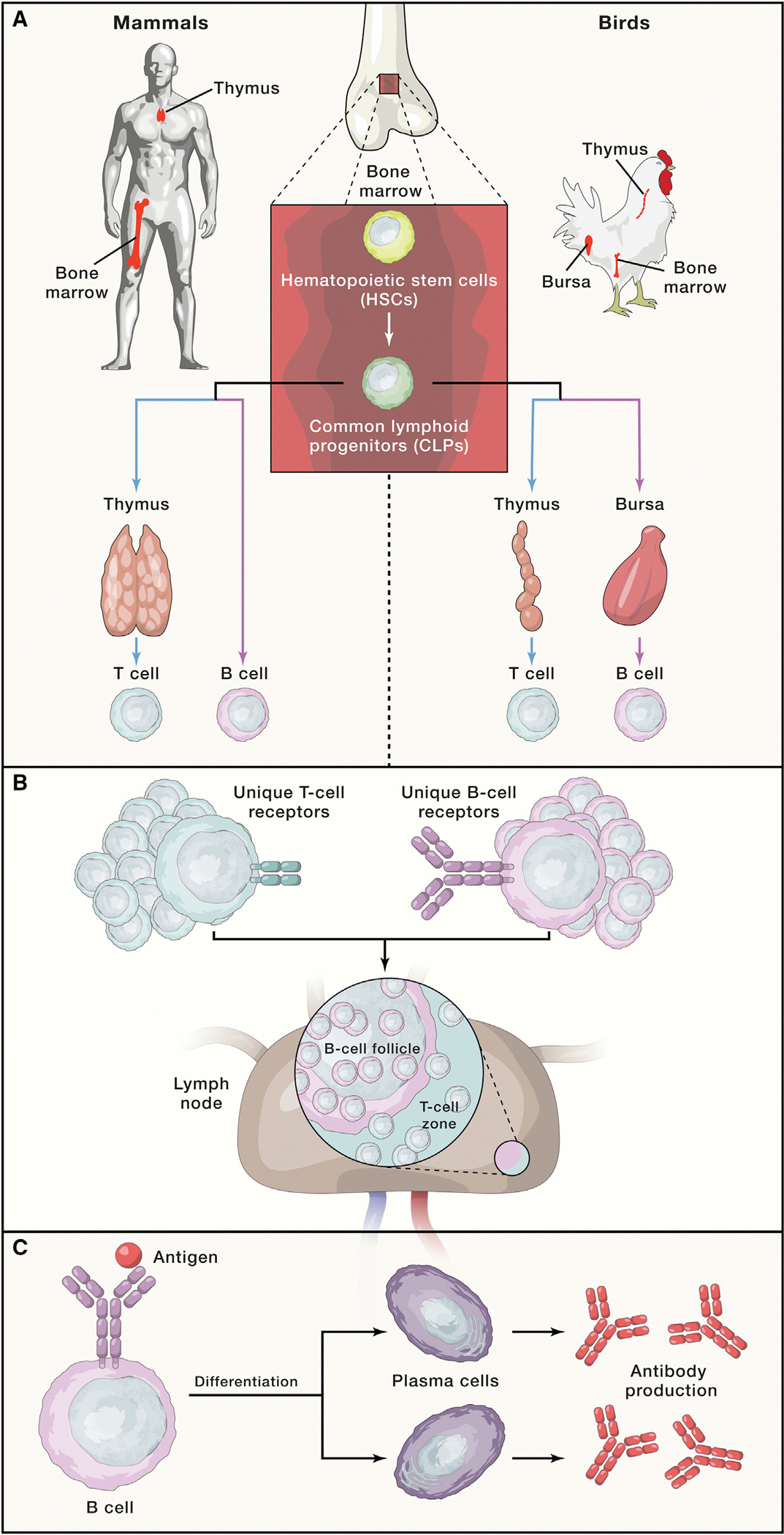
Cooper and Miller’s Discoveries Illuminated Our Understanding of the Dual Arms of the Adaptive Immune System (A) Immature B cells from the chicken bursa of Fabricius, or bone marrow (in mammals), migrate to peripheral lymphoid tissues to mature. Immature T cell precursors emigrate from the bone marrow to the thymus, where they are “educated” for self-tolerance and then leave to seed peripheral tissues. (B) B and T cells occupy distinct anatomical niches in the lymph node and spleen and co-mingle to enable B cells to produce antibodies, known as T cell “help.” (C) Upon antigen encounter, B cells mature into plasma cells and produce antibodies.
